# Multidisciplinary home training for stroke patients in Portugal: the perspective of care professionals

**Published:** 2012-09-04

**Authors:** Silvina Santana, M Viana, Mariana Ribeiro, Conceição Neves

**Affiliations:** Associate Professor with Aggregation, Department of Economics, Management and Industrial Engineering; Institute of Electronics Engineering and Telematics of Aveiro, and Research Unit in Governance, Competitiveness and Public Policies, University of Aveiro, Aveiro, Portugal; Institute of Electronics Engineering and Telematics of Aveiro, Department of Economics, Management and Industrial Engineering, University of Aveiro, Aveiro, Portugal; Institute of Electronics Engineering and Telematics of Aveiro, Department of Economics, Management and Industrial Engineering, University of Aveiro, Aveiro, Portugal; Chiefe Nurse, Unidade de Acidentes Vasculares Cerebrais (Stroke Unit), Hospital Infante Dom Pedro, Aveiro, Portugal

**Keywords:** Home rehabilitation, home-care services, multidisciplinary team, relevance, feasibility, acceptability, stroke, Portugal

## Abstract

**Purpose:**

To systematize the content of home-care interventions for stroke patients in Portugal and assess their relevance, feasibility and acceptability from the perspective of care professionals.

**Theory:**

Research seems to show that Integrated Home Care (IHC) is an excellent form of rehabilitation as the domicile is the place where the patient feels safe, participate most and have direct feedback from ADL training [1]. Moreover, recent results show that IHC has the potential to lower the costs of post-stroke rehabilitation [2]. Cost-effectiveness analysis would then be an important tool for decision-makers regarding alternative IHC interventions. However, in practice the realization of the potential benefits of IHC has to overcome serious barriers and the dissemination of IHC in the European Union will depend “on the upgrade of health professionals from a defensive kind of monodisciplinary professionalism towards an open-ended multidisciplinary professionalism” [1]. Therefore, it is fundamental to assess health professionals’ perceptions and experiences regarding the relevance, feasibility and acceptability of home-based rehabilitation and their willingness to participate.

**Methods:**

In Portugal, the home rehabilitation teams include a gerontologist which is the case manager, a physiotherapist, an occupational therapist and a psychologist. A mixed method was used to analyse the teams’ interventions at home. Information about each session was recorded by all professionals in a standardized form. Additionally, case managers have written qualitative reports, according to a pre-defined minimum structure and the psychologist has written patient specific psychology reports. We analysed the sessions’ reports of 80 patients who have received rehabilitation services at home. This information was coded, keyed into a database and analyzed with PASW Statistics 18. In the qualitative phase, we have explored health professional’s perceptions on experiences with home-care rehabilitation process. Data were collected within a focus group composed by two physiotherapists, two occupational therapists, a psychologist and two gerontologists. A group of three researchers with specific roles joined the focus group, namely a moderator, a recorder and a coordinator. The session’s content was recorded, transcript and analyzed with NVivo 9.

**Results and conclusions:**

Quantitative results consist on the average number of sessions at home per specialty, the duration of sessions, the reasons for therapeutic rehabilitation, the type of intervention executed by the professionals, the specific content of treatments in home care and strategies used by professionals in this type of rehabilitation. Qualitative results include health professionals individual and collective understanding of patients’ achievements and effort required from the professionals and their assessment of the measures used to evaluate the results.

## References

[1] Larsen T (2009). Evidence on the Efficacy of Integrated Care. International Journal of Healthcare Delivery Reform Initiatives.

[2] Larsen T (2011). Home training of patients with stroke—health economic evaluation of a randomised multi centre study performed at three centres in Denmark.

